# *Cryptosporidium proliferans* n. sp. (Apicomplexa: Cryptosporidiidae): Molecular and Biological Evidence of Cryptic Species within Gastric *Cryptosporidium* of Mammals

**DOI:** 10.1371/journal.pone.0147090

**Published:** 2016-01-15

**Authors:** Martin Kváč, Nikola Havrdová, Lenka Hlásková, Tereza Daňková, Jiří Kanděra, Jana Ježková, Jiří Vítovec, Bohumil Sak, Ynes Ortega, Lihua Xiao, David Modrý, Jeba Rose Jennifer Jesudoss Chelladurai, Veronika Prantlová, John McEvoy

**Affiliations:** 1 Institute of Parasitology, Biology Centre of the Czech Academy of Sciences, České Budějovice, Czech Republic; 2 Faculty of Agriculture, University of South Bohemia in České Budějovice, České Budějovice, Czech Republic; 3 Grammar School and High School of Economics, Vimperk, Czech Republic; 4 Faculty of Science, University of South Bohemia in České Budějovice, České Budějovice, Czech Republic; 5 Center for Food Safety, Department of Food Science & Technology, University of Georgia, Griffin, Georgia, United States of America; 6 Centers for Disease Control and Prevention, Atlanta, Georgia, United States of America; 7 Department of Pathology and Parasitology, University of Veterinary and Pharmaceutical Sciences, Brno, Czech Republic; 8 CEITEC VFU, Brno, Czech Republic; 9 Veterinary and Microbiological Sciences Department, North Dakota State University, Fargo, North Dakota, United States of America; Albert Einstein College of Medicine, UNITED STATES

## Abstract

The morphological, biological, and molecular characteristics of *Cryptosporidium muris* strain TS03 are described, and the species name *Cryptosporidium proliferans* n. sp. is proposed. *Cryptosporidium proliferans* obtained from a naturally infected East African mole rat (*Tachyoryctes splendens*) in Kenya was propagated under laboratory conditions in rodents (SCID mice and southern multimammate mice, *Mastomys coucha*) and used in experiments to examine oocyst morphology and transmission. DNA from the propagated *C*. *proliferans* isolate, and *C*. *proliferans* DNA isolated from the feces of an African buffalo (*Syncerus caffer*) in Central African Republic, a donkey (*Equus africanus*) in Algeria, and a domestic horse (*Equus caballus*) in the Czech Republic were used for phylogenetic analyses. Oocysts of *C*. *proliferans* are morphologically distinguishable from *C*. *parvum* and *C*. *muris* HZ206, measuring 6.8–8.8 (mean = 7.7 μm) × 4.8–6.2 μm (mean = 5.3) with a length to width ratio of 1.48 (n = 100). Experimental studies using an isolate originated from *T*. *splendens* have shown that the course of *C*. *proliferans* infection in rodent hosts differs from that of *C*. *muris* and *C*. *andersoni*. The prepatent period of 18–21 days post infection (DPI) for *C*. *proliferans* in southern multimammate mice (*Mastomys coucha*) was similar to that of *C*. *andersoni* and longer than the 6–8 DPI prepatent period for *C*. *muris* RN66 and HZ206 in the same host. Histopatologicaly, stomach glands of southern multimammate mice infected with *C*. *proliferans* were markedly dilated and filled with necrotic material, mucus, and numerous *Cryptosporidium* developmental stages. Epithelial cells of infected glands were atrophic, exhibited cuboidal or squamous metaplasia, and significantly proliferated into the lumen of the stomach, forming papillary structures. The epithelial height and stomach weight were six-fold greater than in non-infected controls. Phylogenetic analyses based on small subunit rRNA, *Cryptosporidium* oocyst wall protein, thrombospondin-related adhesive protein of *Cryptosporidium*-1, heat shock protein 70, actin, heat shock protein 90 (MS2), MS1, MS3, and M16 gene sequences revealed that *C*. *proliferans* is genetically distinct from *C*. *muris* and other previously described *Cryptosporidium* species.

## Introduction

Apicomplexan parasites of the genus *Cryptosporidium* infect the gastrointestinal tract of most vertebrates, including humans [1]. The organ specificity (localization of endogenous development in the host) of *Cryptosporidium* species and genotypes can vary, and two major groups are recognized: the larger intestinal group, which also includes species and genotypes with affinity for the lungs and bursa of Fabricius, and the smaller gastric group which has affinity for the glands of the glandular stomach [2]. Within the gastric group, two species, *C*. *muris* and *C*. *andersoni*, are specific for mammals, and a number of different strains of these species have been identified worldwide (Tables 1 and 2) [3, 4]. *Cryptosporidium muris* was described in laboratory mice [3]. Although it is predominantly a rodent species, it has been detected in, or experimentally transmitted to, various mammalian hosts, including members of Hyracoidea, Carnivora, Lagomorpha, Arctiodactyla, Perissodactyla, and primates (both human and non-human) (Table 1). *Cryptosporidium muris* also has been detected in the feces of snakes, lizards, frogs, and birds of prey; however, these cases were probably due to the mechanical passage of oocysts following ingestion of infected rodents rather than an active infection [5–12]. Similarly, the detection of *C*. *muris* in pig feces and slurry from pig farms [13–15] may have been due to rodents rather than active infections, as pigs are not susceptible to *C*. *muris* under experimental conditions [16]. *Cryptosporidium andersoni*, previously known as *C*. *muris*-like, was distinguished from *C*. *muris* based on molecular and biological differences [4]. Cattle (*Bos taurus*) are the typical host for *C*. *andersoni*, but it has been found in other ruminants, various rodents, and humans (Table 1).

**Table 1:** List of identified hosts for *Cryptosporidium proliferans* (CP), *Cryptosporidium muris* (CM), *Cryptosporidium andersoni* (CA), Japan field mouse genotype (JG), and caribou genotype (CG). Mark indicates susceptibility to infection.

Order	Host (common name)	CP	CM	CA	JG	CG	Reference
**Rodentia**	*Acomys cahirinus* (Spiny mouse)	•	•				[24]
	*Apodemus speciosus* (Large Japanese field mouse)				•		[17]
	*Apodemus sylvaticus* (Wood mouse)		•				[26]
	*Clethrionomys glareolus* (Bank vole)		•				[26]
	*Cavia porcellus* (Guinea pig)		•				[27]
	*Dolichotis patagonum* (Patagonian mara)		•				[10]
	*Eutamias sibiricus* (Siberian chipmunk)		•				[28]
	*Gerbilus gerbilus* (Lesser gerbil)	•		•			[29]
	*Meriones tristrami* (Tristram's jird)	•		•			[29]
	*Meriones unguiculatus* (Mongolian gerbil)	•		•			[21, 30]
	*Sekeetamys calurus* (Bushy-tailed jird)	•		•			[29]
	*Marmota bobak* (Bobak marmot)			•			[31]
	*Mastomys natalensis* (Natal multimammate mouse)	•	•	•			[32]
	*Mastomys coucha* (Southern multimammate mouse)	•	•	•			This study
	*Mesocricetus auratus* (Golden hamster)		•	•			[33, 34]
	*Microtus brandti* (Brandt's voles)	•	•				[24]
	*Mus* spp. (House mouse)	•	•	•	•		[6, 17, 35]
	*Phodopus campbelli* (Campbell's dwarf hamster)		•	•			[33]
	*Phodopus sungorus* (Djungarian hamster)		•	•			[33]
	*Phodopus roborovskii* (Roborovski hamster)		•				[36]
	*Rattus norvegicus* (Brown rat)		•				[37]
	*Sciurus carolinensis* (Eastern gray squirrel)	•					[38]
	*Tachyoryctes splendens* (East African mole-rat)	•					[22]
**Peramelemorphia**	*Macrotis lagotis* (Greater bilby)		•				[39]
**Hyracoidea**	*Procavia capensis* (Rock hyrax)		•				[40]
**Carnivora**	*Canis familiaris* (Dog)		•				[27]
	*Canis latrans* (Coyote)		•				[41]
	*Felis catus* (Cat)		•				[37]
	*Phoca hispida* (Ringed seal)		•				[42]
**Lagomorpha**	*Oryctolagus cuniculus* (European rabbit)		•				[27]
**Arctiodactyla**	*Bos grunniens* (Yak)			•			[43]
	*Bos taurus* (Cattle)			•			[4]
	*Bison bonasus* (European bison)			•			[31]
	*Capra hircus* (Goat)		•				[22, 44]
	*Ovis aries* (Sheep)		•	•			[22, 44, 45]
	*Camelus bactrianus* (Bactrian camel)			•			[46]
	*Gazella cuvieri* (Cuvier's gazelle)		•				[47]
	*Giraffa camelopardalis reticulate* (Reticulated giraffe)		•				[48]
	*Odocoileus hemionus* (Mule deer)						[49]
	*Oreamnos americanus* (Mountain goat)			•			[50]
	*Rangifer tarandus* (Reindeer)					•	[51]
	*Sus scrofa* (Pig)		•				[15]
	*Syncerus caffer* (African buffalo)	•					[52]
**Perissodactyla**	*Equus africanus* (Donkey)	•					[53]
	*Equus ferus caballus* (Horse)	•	•				This study; [54]
**Primates**	*Homo sapiens* (Human)		•	•			[55, 56]
	*Macaca fascicularis* (Crab-eating macaque)		•				[57]
	*Nycticebus coucang* (Sunda slow loris)		•				[58]
**Struthioniformes**	*Struthio camelus* (Ostrich)		•				[59]
**Caprimulgiformes**	*Podargus strigoides* (Tawny frogmouth)		•				[9]
**Galliformes**	*Rollulus roulouli* (Crested partridge)			•			[9]
**Squamata**	*Elaphe obsolete* (Western rat snake)		•				[60]
	*Oxyuranus scutellatus* (Coastal taipan)		•				[6]
	*Python regius* (Python regius)		•				[61]
	*Spilotes pullatus* (Spilotes pullatus)		•				[19]
	*Varanus salvadorii* (Varanus salvadorii)		•				[31]
**Anura**	*Ceratophrys ornate* (Argentine horned frog)		•				[12]

**Table 2:** Prepatent and patent period for *Cryptosporidium proliferans* and different *Cryptosporidium muris* and *Cryptosporidium andersoni* strains and isolates in various hosts.

Species / strain	Original host	Recipient host (age in days)	Prepatent period (day)	Patent period (day)	Infectious dose	Reference
*C*. *muris* MCR	*Mus musculus*	*Mus musculus* ICR (21)	4–10	36–73	2×10^6^	[34, 62]
*C*. *muris* RN66	*Rattus norvegicus*	*Mus musculus* ICR (21)	6	> 30	2.4×10^6^	[63]
*C*. *muris* CB03	*Camelus bactrianus*	*Mus musculus* BALB/c (56)	10	15–18	1×10^6^	[25]
*C*. *andersoni* LI03	*Bos taurus*	*Mus musculus* BALB/c (56)	NI	NI	1×10^6^	[22]
*C*. *proliferans*	*Tachyoryctes splendens*	*Mus musculus* BALB/c (56)	10–12	23–28	1×10^6^	[20]
*C*. *muris* MCR	*Mus musculus*	*Ovis aries* (1–20)	28–35	16–38	2×10^7^	[44]
*C*. *muris* RN66	*Rattus norvegicus*	*Ovis aries* (21)	NI	NI	1×10^7^	[22]
*C*. *muris* CB03	*Camelus bactrianus*	*Ovis aries* (21)	24	< 10	1×10^7^	[22]
*C*. *andersoni* LI03	*Bos taurus*	*Ovis aries* (21)	NI	NI	1×10^7^	[22]
*C*. *proliferans*	*Tachyoryctes splendens*	*Ovis aries* (21)	NI	NI	1×10^7^	[22]
*C*. *muris* MCR	*Mus musculus*	*Capra hircus* (1–20)	19–35	34–85	2×10^7^	[44]
*C*. *muris* RN66	*Rattus norvegicus*	*Capra hircus* (21)	NI	NI	1×10^7^	[22]
*C*. *muris* CB03	*Camelus bactrianus*	*Capra hircus* (21)	28	20–60	1×10^7^	[22]
*C*. *andersoni* LI03	*Bos taurus*	*Capra hircus* (21)	NI	NI	1×10^7^	[22]
*C*. *proliferans*	*Tachyoryctes splendens*	*Capra hircus* (21)	NI	NI	1×10^7^	[22]
*C*. *muris* RN66	*Rattus norvegicus*	*Mus musculus* SCID (28)	6	> 28	1×10^6^	[35]
*C*. *muris* CB03	*Camelus bactrianus*	*Mus musculus* SCID (56)	7	> 60	1×10^6^	Unpublished data
*C*. *andersoni* LI03	*Bos taurus*	*Mus musculus* SCID (56)	NI	NI	1×10^6^	[22]
*C*. *andersoni* Kawatabi	*Bos taurus*	*Mus musculus* SCID (28)	14	> 28	1×10^6^	[35]
*C*. *proliferans*	*Tachyoryctes splendens*	*Mus musculus* SCID (56)	12–18	> 60	1×10^6^	[20]
*C*. *muris* CB03	*Camelus bactrianus*	*Lasiopodomys brandtii*	14	14–32	1×10^6^	[24]
*C*. *andersoni* LI03	*Bos taurus*	*Lasiopodomys brandtii*	NI	NI	1×10^6^	[24]
*C*. *proliferans*	*Tachyoryctes splendens*	*Lasiopodomys brandtii*	14	> 40	1×10^6^	[24]
*C*. *muris* RN66	*Rattus norvegicus*	*Mastomys coucha* (56)	7–8	38–56	1×10^6^	Unpublished data
*C*. *muris* CB03	*Camelus bactrianus*	*Mastomys coucha* (56)	8–10	51–76	1×10^6^	Unpublished data
*C*. *andersoni* LI03	*Bos taurus*	*Mastomys coucha* (56)	20	46–59	1×10^6^	[64]
*C*. *proliferans*	*Tachyoryctes splendens*	*Mastomys coucha* (56)	15–20	> 140	1×10^6^	This study
*C*. *muris* HZ206	*Mus musculus*	*Mastomys coucha* (56)	6–8	48–77	1×10^6^	This study
*C*. *andersoni*	*Bos taurus*	*Meriones unguiculatus* (56)	15–19	18–65	1×10^6^	[30]
*C*. *proliferans*	*Tachyoryctes splendens*	*Meriones unguiculatus* (56)	18–22	> 90	1×10^6^	[21]

**NI**–non infectious

A number of variant strains of *C*. *muris* and *C*. *andersoni* have been described, based on polymorphisms in the small ribosomal subunit (SSU) gene, and differences in host specificity, pathogenicity, and course of infection. For example, *C*. *muris* Japan field genotype (also known as *C*. *muris* Kawatabi strain) differs from *C*. *muris* RN66 (reference strain) [17, 18]. Similarly, among *C*. *andersoni* strains, only *C*. *andersoni* Kawatabi, is infectious for the domestic mouse. Previous studies suggest that *C*. *muris* and *C*. *andersoni* represent a complex of cryptic species (Tables 1 and 2), but phylogenetic and biological data to support separate species are mostly lacking.

We undertook this study to examine the host specificity, course of infection, pathogenicity, oocyst morphology, and molecular characteristics of C. muris strain TS03. Based on the collective data from this and other studies [19–25], which show that C. muris strain TS03 is genetically distinct from C. muris and other known Cryptosporidium species, we propose the species name Cryptosporidium proliferans n. sp.

## Materials and Methods

### Source of oocysts and DNA for studies

The isolate of *C*. *proliferans *(previously known as *C*. *muris* TS03) used to determine experimental infectivity and oocyst morphology originated from a naturally infected East African mole rat (*Tachyoryctes splendens*) trapped in Kakamega, Kenya in 2003, and was maintained in susceptible laboratory rodents (SCID and southern multimammate mice *Mastomys coucha*) at two laboratories: Institute of Parasitology, Biology Centre of the Academy of Sciences of the Czech Republic and University of Veterinary and Pharmaceutical Sciences Brno, Czech Republic. DNA obtained from the laboratory-propagated *C*. *proliferans* isolate, and *C*. *proliferans* DNA isolated from the feces of an African buffalo (*Syncerus caffer*) in Central African Republic [52], a donkey (*Equus africanus*) in Algeria [53], and a domestic horse (*Equus caballus*) in the Czech Republic (unpublished) were used for phylogenetic analyses.

Oocysts of *C*. *muris* HZ206, originally isolated from a naturally infected domestic mouse (*Mus musculus domesticus*; Mmd) in Germany in 2012, were used as a *C*. *muris* control. *Cryptosporidium muris* HZ206 has been maintained at the Institute of Parasitology, Biology Centre of the Academy of Sciences of the Czech Republic in a wild-derived Mmd strain from Schweben, central Germany (10th generation of brother-sister mating; kept under the name SCHEST at the Institute of Vertebrate Biology, Brno, Czech Republic). For comparison of oocyst morphology a *C*. *parvum* isolate originating from a naturally infected 23-day-old Holstein calf was used.

### Parasitological examination and oocyst preparation

Animal feces were screened for *Cryptosporidium* oocysts using fecal smears stained with aniline-carbol-methyl violet (ACMV) [65]. Fecal specimens were collected daily and stored in a 2.5% potassium dichromate solution at 4–8°C. *Cryptosporidium* oocysts were purified for morphometry, phylogeny, and infectivity analyses using sucrose gradient [66] and cesium chloride gradient centrifugation [67]. Purified oocysts were stored for up to 4 weeks at 4–8°C in PBS with antimycotics and antibiotics (100 UI penicillin, 10 μg/ml streptomycin, 0.25/ml μg amphotericin and 30 μg/ml gentamicin). The identity of the parasite was confirmed by sequence analysis of the SSU gene, using the method described below. The number of oocysts administered to animals was determined by hemocytometer counting. The viability of oocysts was examined by propidium iodide (PI) staining using a modification of a previously described assay [68]. Examined oocysts were washed in distilled water (DW; 10^5^ oocysts in 100 μl) and mixed with 10 μl of PI (1% solution, SIGMA). After 30 min of incubation at room temperature in the dark, the oocysts were washed twice with DW. Oocyst viability was examined using fluorescence microscopy (filter 420 nm, Olympus IX70). Oocysts with red fluorescence were considered to be dead, and those without fluorescence were considered viable. A total of 500 oocysts were counted.

### Oocyst morphology

Oocysts were examined using differential interference contrast (DIC) microscopy following ACMV and Auramine Phenol (AP) staining [69], or fluorescence microscopy following labeling with genus-specific FITC-conjugated antibodies (*Cryptosporidium* IF Test, Crypto cel, Medac) (Olympus IX70 microscope; Olympus CZ, Czech Republic). Morphology and morphometry were determined using digital analysis of images (M.I.C. Quick Photo Pro v.3.0 software; Optical Service, Czech Republic) collected using an Olympus Digital Colour Camera DP73 (17.29 megapixels). A 20-μl aliquot containing 10^5^ purified oocysts was examined for each measurement. Length and width of oocysts (n = 100) were measured under DIC at 1000× magnification, and these were used to calculate the shape index and length-to-width ratio of each oocyst. As a control, the morphometry of *C*. *parvum* (n = 100) from a naturally infected 23-day-old Holstein calf, and *C*. *muris* HZ206 (n = 100) were measured. Photomicrographs of *C*. *proliferans* oocysts observed by DIC, ACMV, AP and IFA were deposited as a phototype at the Institute of Parasitology, Biology Centre of the Academy of Sciences of the Czech Republic.

### DNA extraction and molecular analyses

Total DNA was extracted from 200 mg of feces, 10^5^ purified oocysts, or 200 mg of tissue by bead disruption for 60 s at 5.5 m/s using 0.5 mm glass beads in a FastPrep®24 Instrument (MP Biomedicals, CA, USA) followed by isolation/purification using a commercially available kit in accordance with the manufacturer’s instructions (QIAamp® DNA Stool Mini Kit or DNeasy® Blood & Tissue Kit, Qiagen, Hilden, Germany). Purified DNA was stored at −20°C prior to being used for PCR. A nested PCR approach was used to amplify ∼830 bp of the small ribosomal subunit (SSU) gene [70, 71], ∼1066 bp of the actin gene [72], and four previously described minisatellite genes—MS1 (encoding a hypothetical protein), MS2 (encoding a 90-kDa heat shock protein), MS3 (encoding a hypothetical protein), and MS16 (encoding a leucine-rich repeat family protein) [19]. Both primary and secondary PCR reactions were carried out in a volume of 50 μl; the primary reaction contained 2 μl of genomic DNA (or water as a negative control) and the secondary reaction contained 2 μl of the primary reaction as template. In addition, primers for nested PCR to amplify Thrombospondin-Related Adhesive Protein of *Cryptosporidium*-1 (TRAP-C1; ~955 bp), *Cryptosporidium* Oocyst Wall Protein (~ 400 bp), and Heat shock protein (HSP70; ~515 bp) were designed for this study using PrimerQuest online software (IDT, http://www.idtdna.com/) and tested by software Serial Cloner v 2.6.1. (http://serialbasics.free.fr/Serial_Cloner.html). Primers amplifying TRAP-C1 were designed using the *C*. *muris* sequence in GenBank (CMU_020100). The primers for primary reactions were TRAP-G-F1 (GGA GAT CCT TTA TGT GTT G) and TRAP-G-R1 (CCT GTA CAA ATT CTT CTG AT) and secondary reaction TRAP-G-F2 (GCT CAG AAG ATC CAA GTA) and TRAP-G-R2 (GAT TGC TCT GAA CTA GGA). Primers amplifying HSP70 gene were designed using the *C*. *muris* sequence in GenBank (CMU_009950). The primers for primary reactions were HSPAvA1-F (GCT CGT GGT CCT AAA GAT AA) and HSPAvA1-R (ACG GGT TGA ACC ACC TAC TAA T) and secondary reaction HSPAvA2-F (ACA GTT CCT GCC TAT TTC A) and HSPAvA2-R (GCT AAT GTA CCA CGG AAA TAA). Primers amplifying the COWP gene were designed using the consensus of sequences in GenBank (B471649, AB471650, KF747672, DQ989571, DQ989570, DQ060431, KF419210, AF266275, AF161580, DQ060430, AF266262, AY282693, AF161579, AF266264, AB514044, AB514043, AY643491, AB089289, and AB089287). The primers for primary reactions were COWP-torto-F1 (GAA TGT CCT CCT GGG ACT GTA) and COWP-torto-R1 (AGT TCC TGG TGG ACA TAC TG) and secondary reaction COWP-torto-F2 (TCC TCC TGG GAC TGT ATT GGA) and COWP-torto-R2 (GGT GGA CAT ACT GGT TGT GTT G). The primary PCR reactions, for TRAP-C1, HSP70, and COWP genes, were carried out in a volume of 50 μl containing 2 μl of DNA template (or water as a negative control), 1×PCR buffer, 3 mM MgCl_2_, 200 μM dNTPs, 0.2 μM of each primer, and 1.5 U of Taq DNA polymerase (Top Bio, Czech Republic). Secondary reactions were carried out under similar conditions with 2 μl of primary product used as template. PCRs were run in a thermo cycler with an initial denaturation of 94°C for 5 min, followed by 35 cycles of 94°C for 45 s, 55°C (TRAP-C1), 50°C (HSP70 and COWP) for 45 s, 72°C for 1 min. A final elongation step of 72°C for 10 min was included to ensure complete extension of amplified products. Conditions were the same for both primary and secondary reactions. DNA of *C*. *andersoni* was used as positive control. Secondary PCR products were detected by agarose gel (2.0%) electrophoresis, visualized by ethidium bromide staining (0.2 μg/ml) and extracted using QIAquick® Gel Extraction Kit (Qiagen). Purified secondary products were sequenced in both directions with an ABI 3130 genetic analyser (Applied Biosystems, Foster City, CA) using the secondary PCR primers and the BigDye1 Terminator V3.1 cycle sequencing kit (Applied Biosystems, Foster City, California) in 10 μl reactions. Amplification and sequencing of each locus was repeated three times.

### Phylogenetic analyses

The nucleotide sequences of each gene obtained in this study were edited using the ChromasPro 1.7.5 software (Technelysium, Pty, Ltd.), manually edited, and aligned with each other and with reference sequences from GenBank using MAFFT version 7 online server with automatic selection of alignment mode (http://mafft.cbrc.jp/alignment/software/). Phylogenetic analyses were performed and best DNA/Protein phylogeny models were selected using the MEGA6 software [73, 74]. Phylogenetic trees were inferred by the **i)** neighbor-joining (NJ), **ii)** maximum likelihood (ML), and **iii)** maximum parsimony (MP) method. Bootstrap support for branching was based on 1000 replications. Neighbor-joining phylograms were edited for style using CorelDrawX7. Sequences have been deposited in GenBank under the accession numbers KR090615-KR090632 and KT731193-KT731212.

### Transmission studies

#### Animals

The infectivity and pathogenicity of C. proliferans for the eight-week-old southern multimammate mice (Mastomys coucha), and adult budgerigars (Melopsittacus undulatus) (Institute of Parasitology, Biology Centre of the Academy of Sciences of the Czech Republic, Czech Republic) was determined experimentally in this study. The infectivity of C. proliferans for SCID and BALB/c mice, gerbils, calves, kids, and lambs to C. proliferans (previously named C. muris strain TS03) was determined previously (Table 2) [21, 22, 25].

#### Experimental design

To prevent environmental contamination with oocysts, southern multimammate mice were housed in plastic cages with sterilized wood-chip bedding situated in flexible film isolators (BEM, Znojmo, Czech Republic) with high-efficiency particulate air filters. Birds were kept in cages placed in a room separated from other animals. The southern multimammate mice and budgerigars were supplied with a standard sterilized diet for rodents and birds, respectively, and sterilized water *ad libitum*. For three week prior to infection, fecal samples from all animals were screened daily for the presence of *Cryptosporidium* spp. using parasitological and molecular tools as described in previous sections. Each animal was inoculated orally by stomach tube with 10^6^ purified viable oocysts of each species (*C*. *proliferans* and *C*. *muris* HZ206) suspended in 200 μl of distilled water. Each animal used as negative control was inoculated with 200 μl of distilled water only. A total of nine budgerigars were used: three as negative controls, three infected with *C*. *proliferans*, and three infected with *C*. *muris* HZ206. Fecal samples from all experimental birds were collected daily and experiments were terminated 30 days post infection (DPI). A total of 94 southern multimammate mice were divided into three groups: i) control group (n = 34), ii) group infected with *C*. *muris* HZ206 (n = 30), and iii) group infected with *C*. *proliferans* (n = 30). All animals were weighed before the start of the experiment, with a precision of 0.1 g. Each animal was kept in separate cages. Fecal samples from all experimental southern multimammate mice were taken daily for the first 28 days, then every 7 days. Experiments were terminated 140 DPI. Animals were euthanized via cervical dislocation (according Law of the Czech Republic No. 419/2012 Sb.). Fecal samples from all experimental animals were stained by ACMV and the presence of *Cryptosporidium* specific DNA was confirmed using nested PCR targeting the SSU gene every 7 days. Every 28 days, 4 southern multimammate mice were sacrificed from each group, and each was examined for body weight, stomach size and weight, and the surface ratio of glandular to non-glandular parts of the stomach. Histopathological changes of gastric mucosa due to infection of *C*. *proliferans* and *C*. *muris* were evaluated using histological methods. Results were compared to uninfected animals, which were tested using the same procedures. Course of infection indicators, including fecal consistency, fecal color and infection intensity, were examined. Infection intensity was reported as the number of oocysts per gram (OPG) of feces as previously described [64].

#### Clinical and histopathological examinations

A complete examination of all gastrointestinal organs was conducted at necropsy. Tissue samples from the stomach, small intestine, and large intestine (the entire tract was divided into 1 cm sections) were processed for histology [75] and for PCR analyses (see Section 2.4.). Histology sections were stained with hematoxylin and eosin (HE), Wolbach’s modified Giemsa, Periodic Acid Schiff (PAS) stain, and genus-specific FITC conjugated monoclonal antibodies targeting *Cryptosporidium* oocyst wall antigens (*Cryptosporidium* IF Test, Crypto Cel, Medac).

### Animal care

Animal caretakers wore disposable coveralls, shoe covers, and gloves every time they entered the facility rooms. All wood-chip bedding, feces, and disposable protective clothing were sealed in plastic bags, removed from the buildings and incinerated. All housing, feeding, and experimental procedures were conducted under protocols approved by the Institute of Parasitology, Biology Centre of the Academy of Sciences of the Czech Republic and Institute and National Committees (Protocols No. 52/2014).

### Statistical analyses

The hypothesis tested in the analysis of oocyst morphometry and size of stomach was that two-dimensional mean vectors of measurement are the same in the two populations being compared. Hotelling’s T2 test was used to test the null hypothesis. The Bartlett test was used to test homoscedasticity of differences in the prepatent and patent periods of different infections.

## Results

### Oocyst morphology

**Fig 1:**
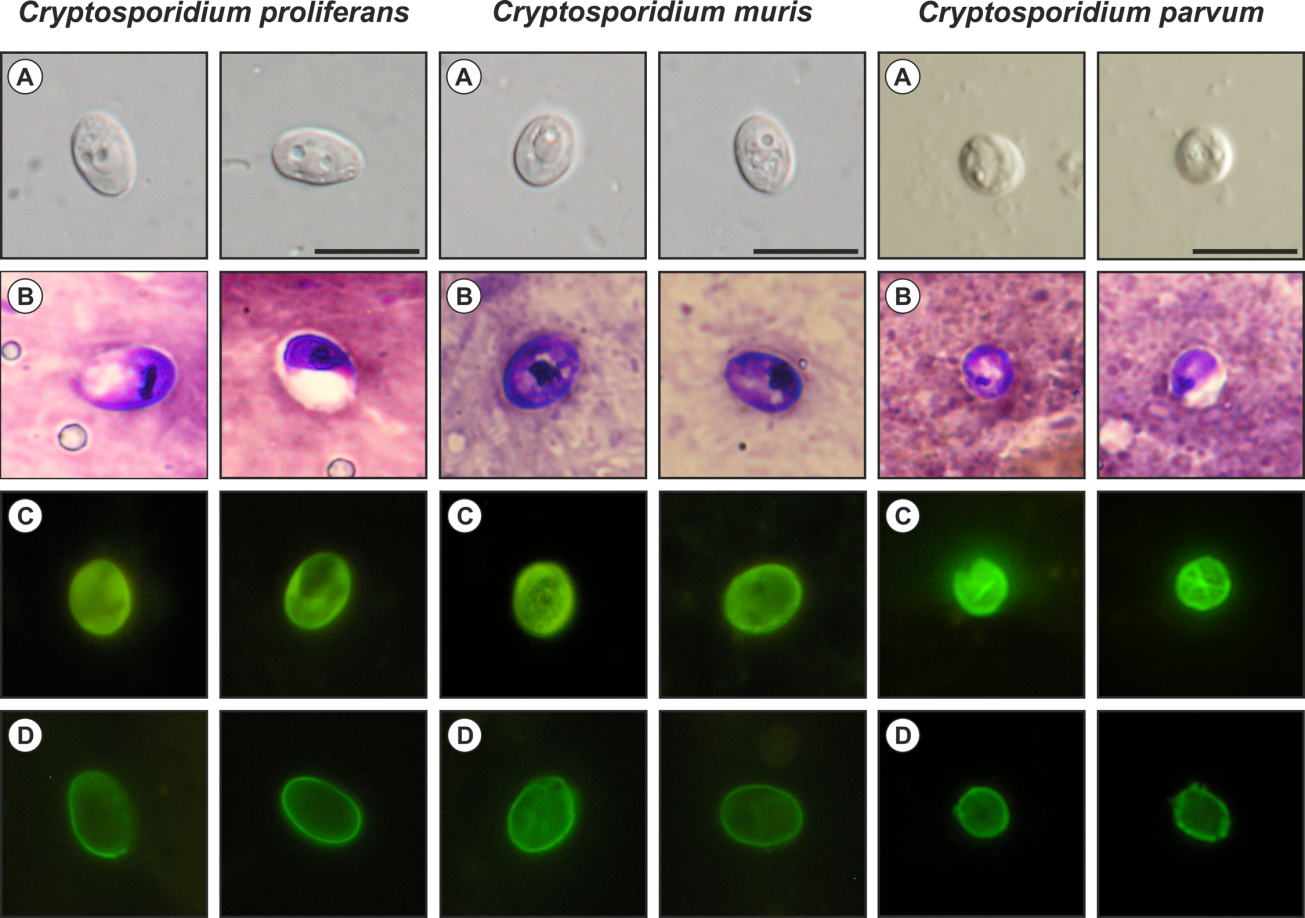
*Cryptosporidium proliferans*, *Cryptosporidium muris* HZ206, and *Cryptosporidium parvum* oocysts in (A) differential interference contrast microscopy and stained by (B) aniline–carbol–methyl violet (C) Auramine Phenol and (D) anti-*Cryptosporidium* FITC-conjugated antibody. Bar = 10 μm.

Oocysts of *C*. *proliferans* measuring 6.8–8.8 (mean = 7.7 μm) × 4.8–6.2 μm (mean = 5.3) with a length to width ratio of 1.48 (n = 100) were significantly longer and narrower (p <0.05) than *C*. *muris* HZ206 oocysts, which measured 6.3–8.1 (mean = 7.5 μm) × 5.0–6.6 (mean = 5.7 μm) with length to width ratio of 1.35 (n = 100). *Cryptosporidium parvum* oocysts were significantly smaller (p<0.05) than *C*. *proliferans* and *C*. *muris* HZ206, measuring 5.1–5.5 (mean = 5.3 μm) × 4.6–5.2 (mean = 4.7 μm) with length to width ratio of 1.12 (1.07–1. 32) (n = 100) (Fig 1A). Oocysts of *C*. *proliferans* recovered from experimentally infected mice were morphologically similar to those used for infection. Oocysts in fecal smears showed typical *Cryptosporidium* ACMV and AP staining characteristics (Fig 1B and 1C). Fixed *C*. *proliferans* oocysts labeled with FITC conjugated anti-*Cryptosporidium* oocyst wall antibody and examined by epifluorescence microscopy displayed typical apple green, halo-like fluorescence (Fig 1D).

### Molecular characterization

**Fig 2:**
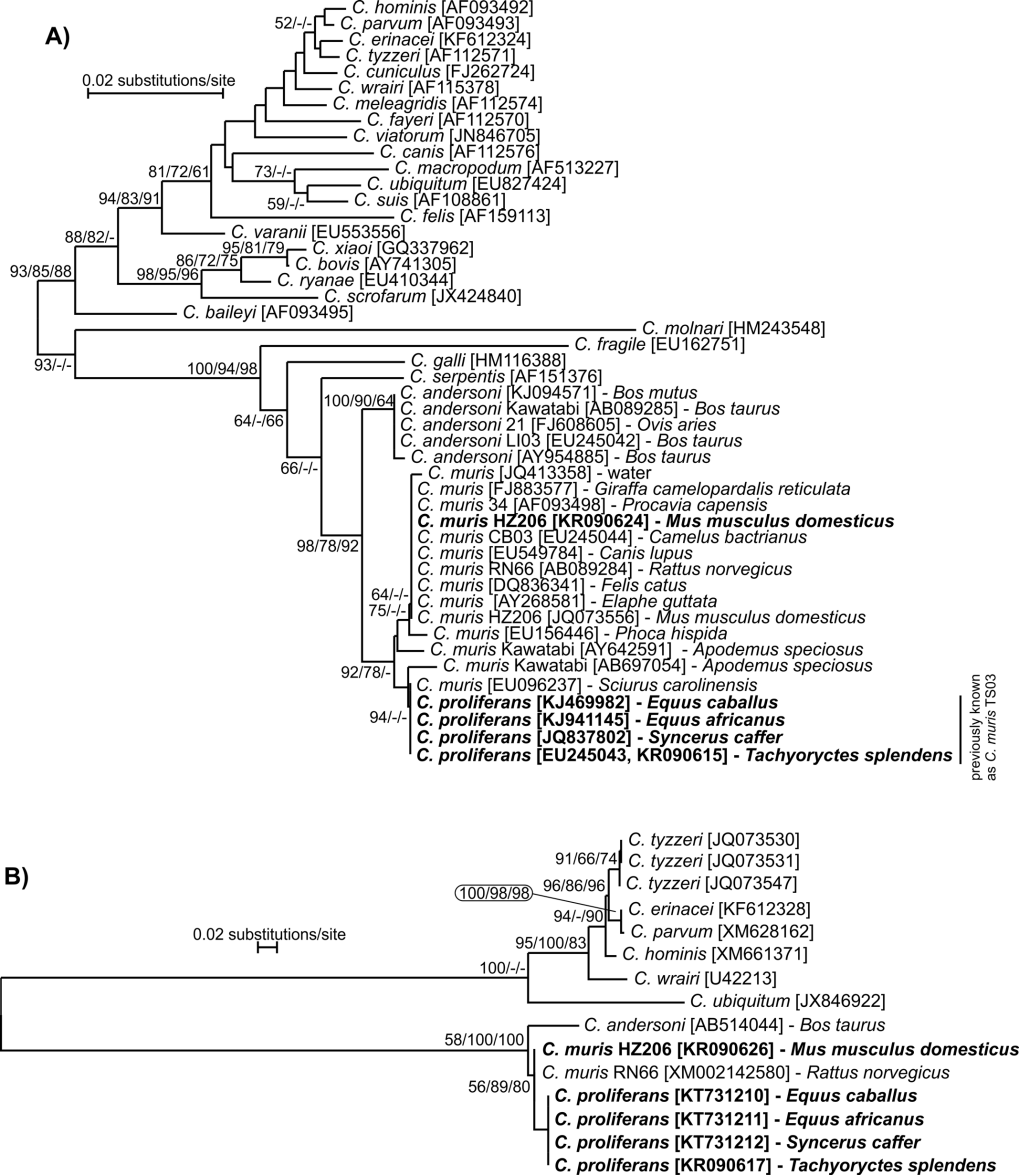
Phylogenetic relationships between *Cryptosporidium proliferans* (highlighted) and other *Cryptosporidium* spp. as inferred by a neighbor-joining analysis (NJ)/maximum parsimony(MP)/maximum likelihood (ML) of (A) the SSU (706 base positions in the final dataset; ML = log -2886.67) and (B) TRAP-C1 (531 base positions in the final dataset, ML = log -1929.25). The percentage of replicate trees in which the associated taxa clustered together in the bootstrap test (1000 replicates). Numbers at the nodes represent bootstrap values for the nodes gaining more than 50% support. Scale bar included in each tree.

At the SSU locus, *C*. *proliferans* isolates from an East African mole rat, African buffalo, donkey, and domestic horse shared 100% identity with each other and with an isolate (EU096237) from an Eastern gray squirrel in the USA (Fig 2A). At the TRAP-C1 locus, isolates of *C*. *proliferans* from the four different hosts shared 100% identity with each other and differed from *C*. *muris* RN66 by five SNPs, two of which were non-synonymous (Fig 2B).

**Fig 3:**
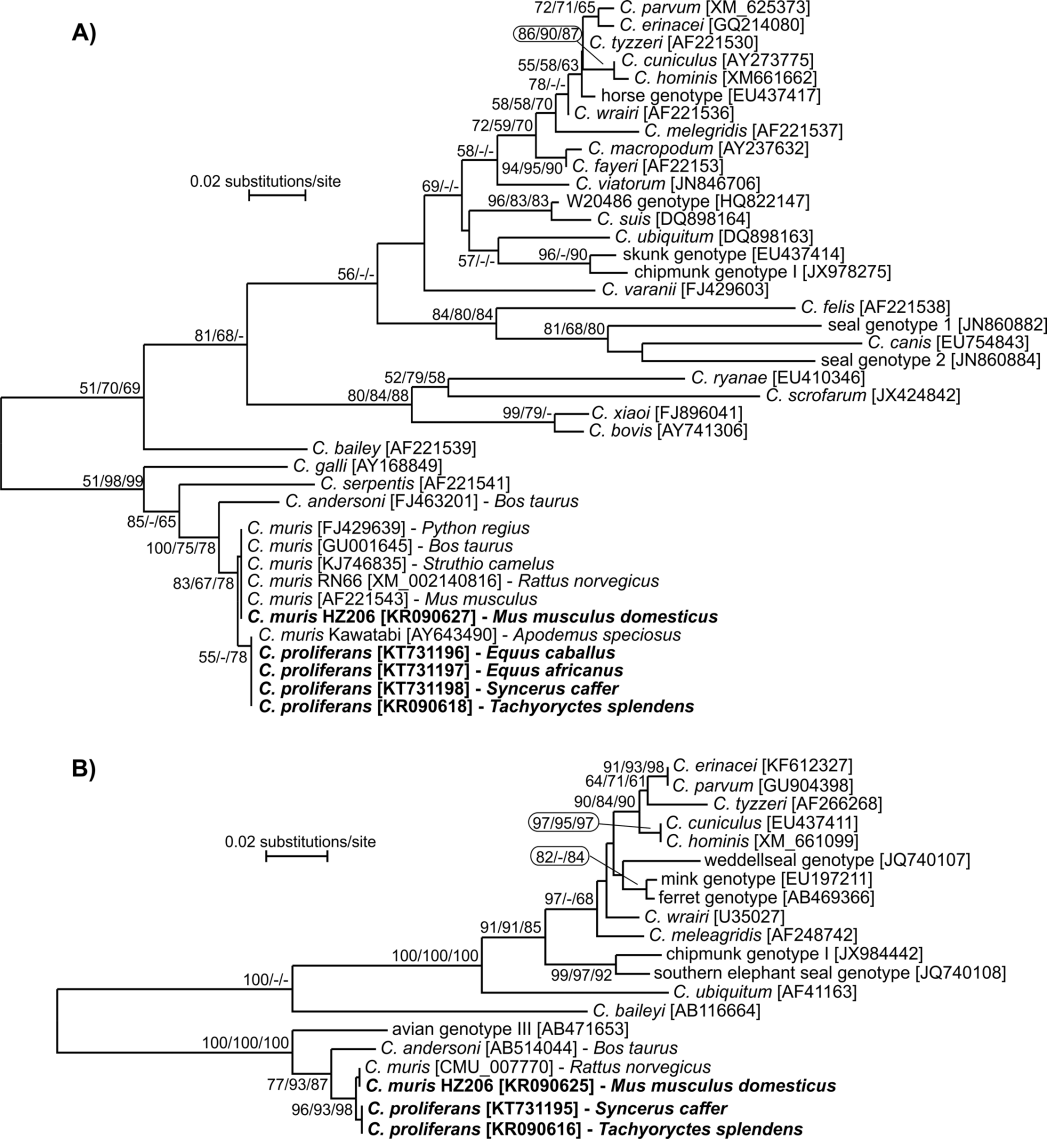
Phylogenetic relationships between *Cryptosporidium proliferans* (highlighted) and other *Cryptosporidium* spp. as inferred by a neighbor-joining analysis (NJ)/maximum parsimony(MP)/maximum likelihood (ML) of (A) HSP70 (211 base positions in the final dataset, ML = log -1745.42) and (B) COWP (369 base positions in the final dataset, ML = log -532.78). The percentage of replicate trees in which the associated taxa clustered together in the bootstrap test (1000 replicates). Numbers at the nodes represent bootstrap values for the nodes gaining more than 50% support. Scale bar included in each tree.

At the HSP70 locus, *C*. *proliferans* isolates from the four different hosts shared 100% identity with each other and with the *C*. *muris* Kawatabi isolate (AY643490) from *Apodemus speciosus* in Japan (Fig 3A), but differed from *C*. *muris* RN66 by a synonymous SNP.

Isolates of *C*. *proliferans* from the East African mole rat and African Buffalo shared 100% identity at the COWP locus, and differed from *C*. *muris* RN66 (CMU_007770) by a synonymous SNP (C/T) at position 597, using the *C*. *parvum* Iowa isolate as a reference sequence (Cgd6_2090) (Fig 3B). *Cryptosporidium proliferans* COWP sequences were not obtained from donkey or domestic horse isolates.

**Fig 4:**
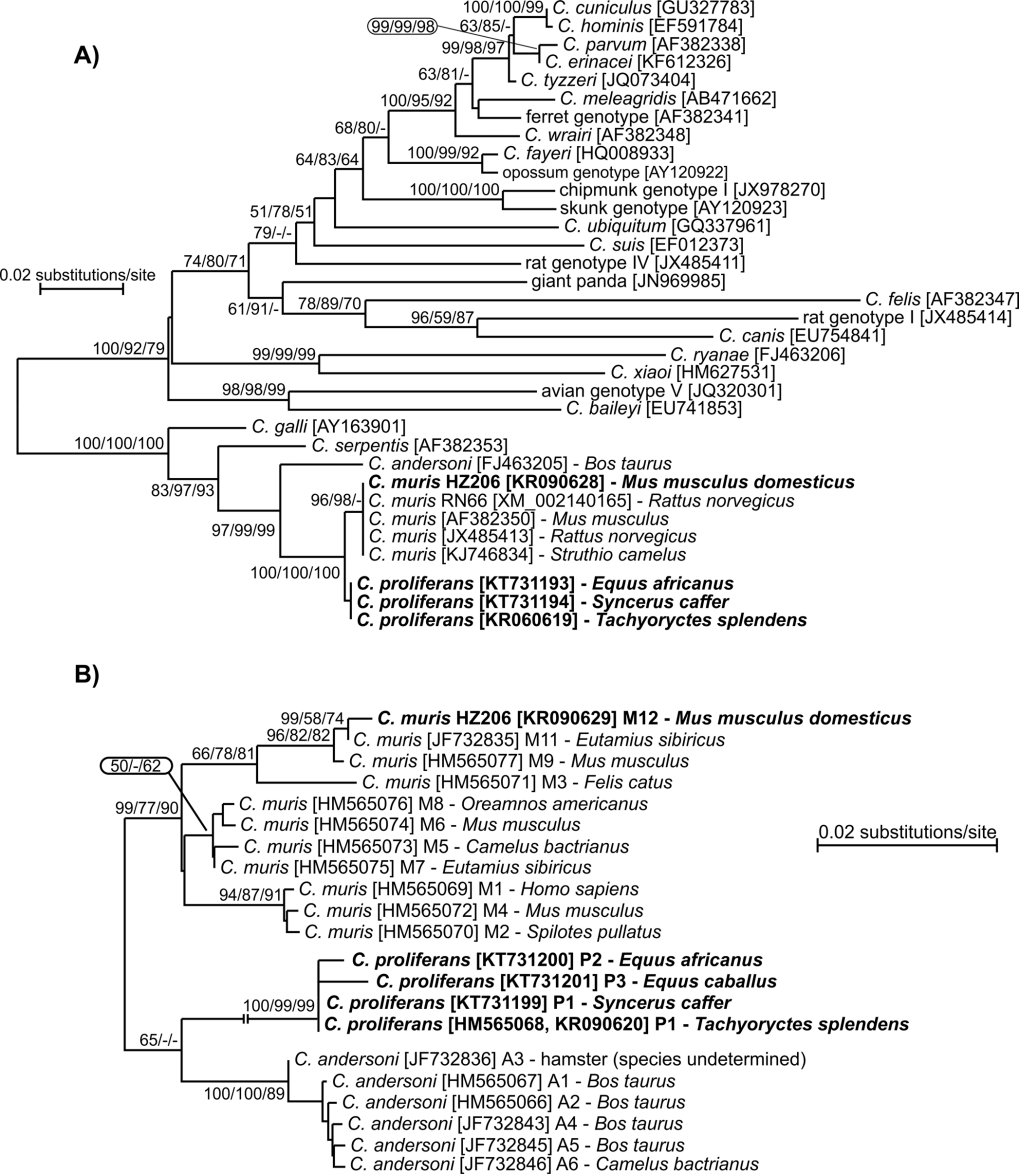
Phylogenetic relationships between *Cryptosporidium proliferans* (highlighted) and other *Cryptosporidium* spp. as inferred by a neighbor-joining analysis (NJ)/maximum parsimony (MP)/maximum likelihood (ML) of (A) actin (728 base positions in the final dataset, ML = log = -5522.35) and (B) MS1 (436 base positions in the final dataset, ML = log -886.75). The percentage of replicate trees in which the associated taxa clustered together in the bootstrap test (1000 replicates). Numbers at the nodes represent bootstrap values for the nodes gaining more than 50% support. Interrupted branches have been shortened five-fold. Scale bar included in each tree.

At the actin locus, isolates from the East African mole rat, African buffalo, and donkey shared 100% identity with each other and differed from *C*. *muris* by four synonymous SNPs (Fig 4A). An actin sequence was not obtained from the domestic horse isolate.

**Fig 5:**
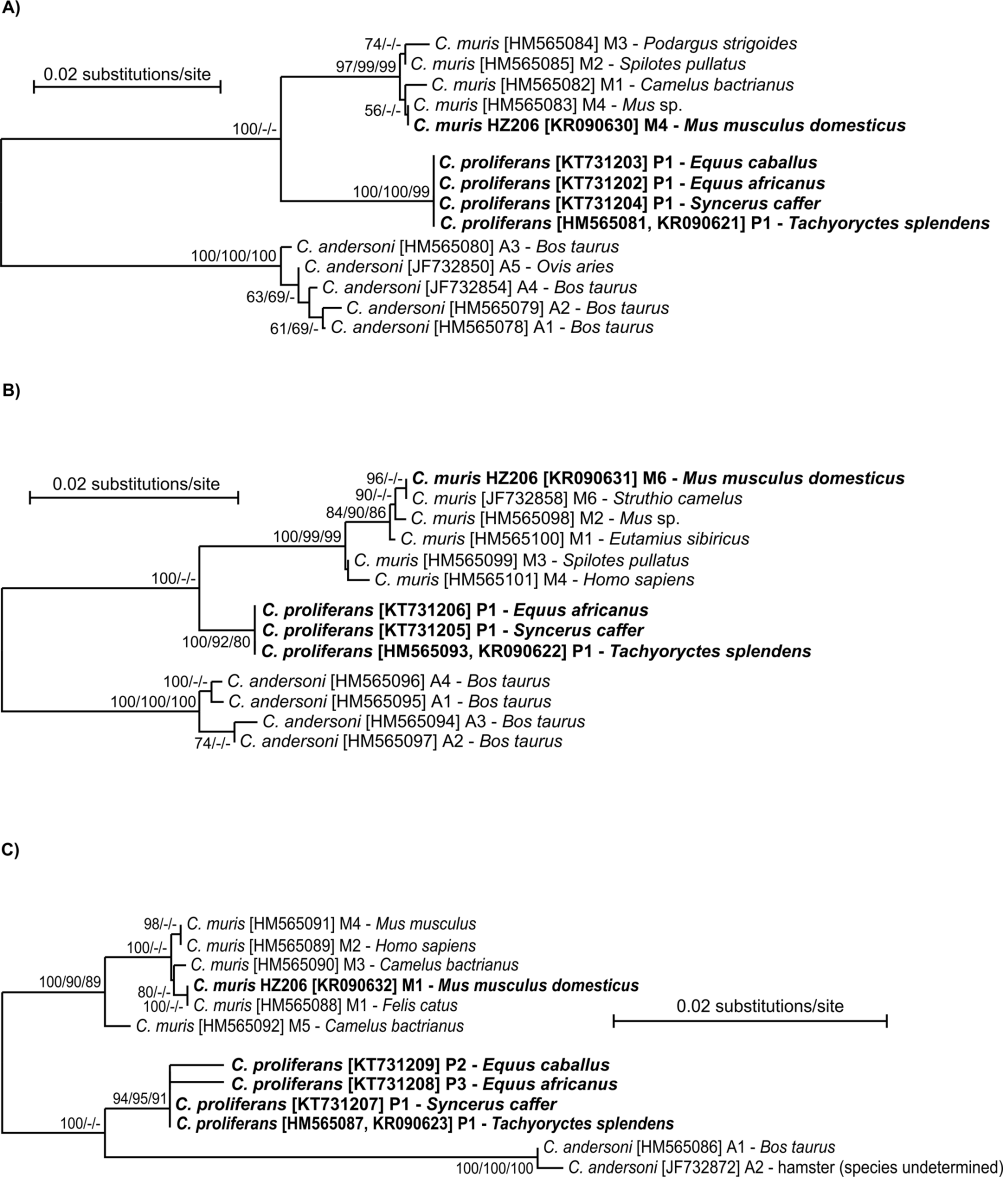
Phylogenetic relationships between *Cryptosporidium proliferans* (highlighted) and other *Cryptosporidium* spp. as inferred by a neighbor-joining analysis (NJ)/maximum parsimony (MP)/maximum likelihood (ML) of (A) MS2 (442 base positions in the final dataset, ML = log -792.33), (B) MS3 (485 base positions in the final dataset, ML = log = -832.45), and (C) MS16 (580 base positions in the final dataset, ML = log = -956.78). The percentage of replicate trees in which the associated taxa clustered together in the bootstrap test (1000 replicates). Numbers at the nodes represent bootstrap values for the nodes gaining more than 50% support. Scale bar included in each tree.

*Cryptosporidium proliferans* isolates clustered separately from *C*. *muris* and *C*. *andersoni* at each of the four microsatellite loci examined. Three *C*. *proliferans* subtypes formed a single cluster at the MS1 locus: MS1-P1 was detected in isolates from the East African mole rat and African Buffalo; MS1-P2 and MS1-P3 were detected in the donkey and domestic horse, respectively (Fig 4B). A single *C*. *proliferans *subtype was detected at the MS2 (MS2-P1) and MS3 loci (MS3-P1) (Fig 5A and 5B). Similar to MS1, three *C*. *proliferans* subtypes formed a single cluster at the MS16 locus: MS16-P1 was detected in isolates from the East African mole rat and African Buffalo; MS16-P2 and MS16-P3 were detected in the domestic horse and donkey, respectively (Fig 5C).

**Fig 6:**
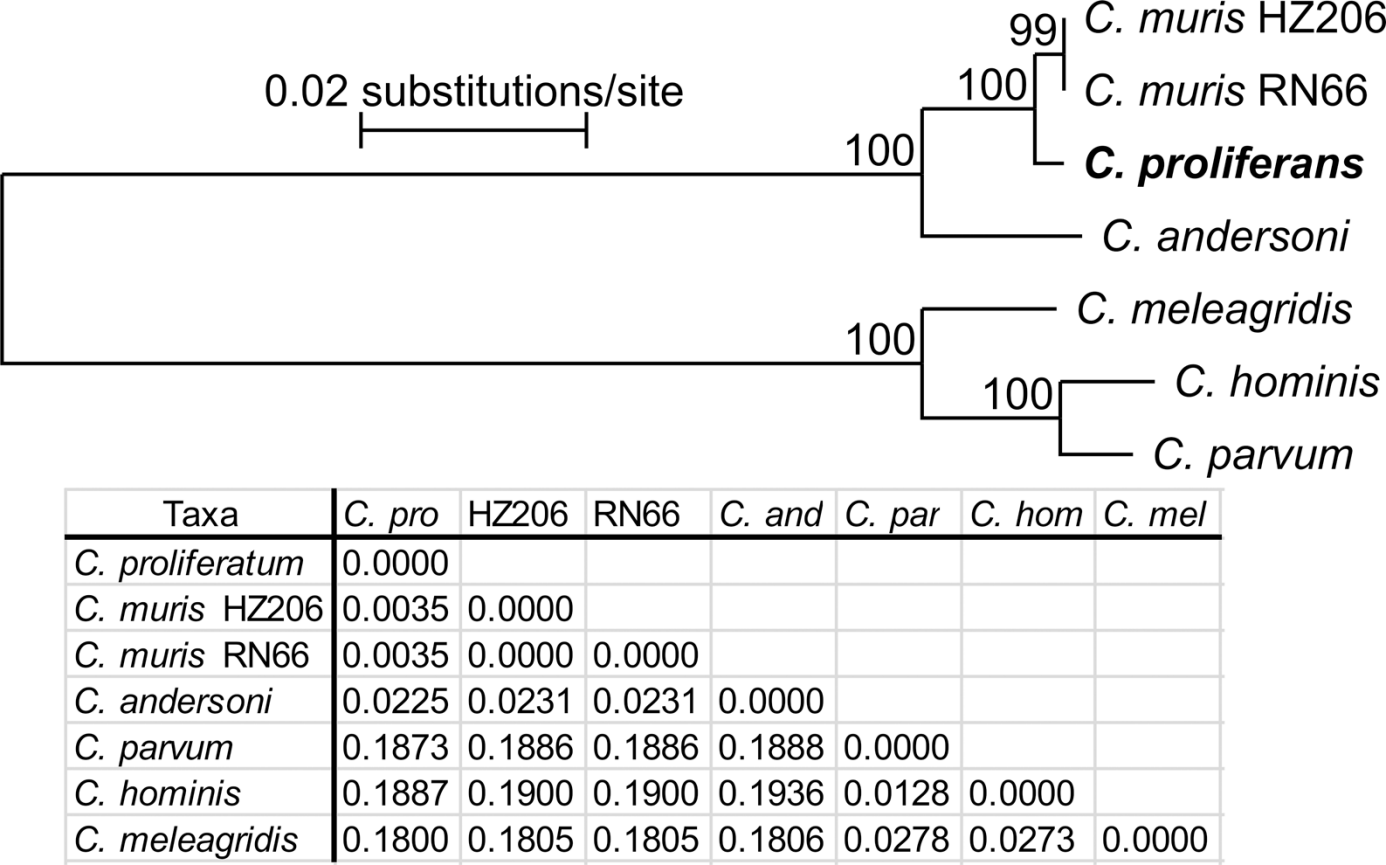
Phylogenetic relationships between *Cryptosporidium proliferans* and selected *Cryptosporidium* spp. as inferred by a neighbor-joining analysis (NJ)/maximum parsimony(MP)/maximum likelihood (ML) analysis of a concatenated sequence constructed from partial DNA sequences of SSU, actin, COWP, HSP70, and TRAP-C1 genes (1991 base positions in the final dataset, ML = log = -4368.98). The percentage of replicate trees in which the associated taxa clustered together in the bootstrap test (1000 replicates). Numbers at the nodes represent bootstrap values for the nodes gaining more than 50% support. Scale bar included in tree.

In a tree constructed from concatenated sequences of SSU, actin, HSP70, and TRAP-C1, *C*. *proliferans* clustered separately from *C*. *muris* RN66, *C*. *muris* HZ206, *C*. *parvum*, *C*. *hominis*, and *C*. *meleagridis* (Fig 6).

### Experimental transmission studies

Oocysts used for experimental infections had >95% viability, determined by PI staining. Experimentally inoculated budgerigars did not produce microscopically detectable infection. Histological and molecular examination of gastrointestinal tract tissue from budgerigars did not reveal the presence of *Cryptosporidium* developmental stages.

Both *C*. *proliferans* and *C*. *muris* HZ206 were infectious for southern multimammate mice. While southern multimammate mice began shedding *C*. *muris* HZ206 oocysts 6–8 DPI, *C*. *proliferans* shedding began significantly later at 18–21 DPI (p<0.001). PCR amplification of the *Cryptosporidium* SSU gene was unsuccessful from 2 to 5 DPI and 2 to 17 DPI in mice infected with *C*. *muris* HZ206 and *C*. *proliferans*, respectively.

**Fig 7:**
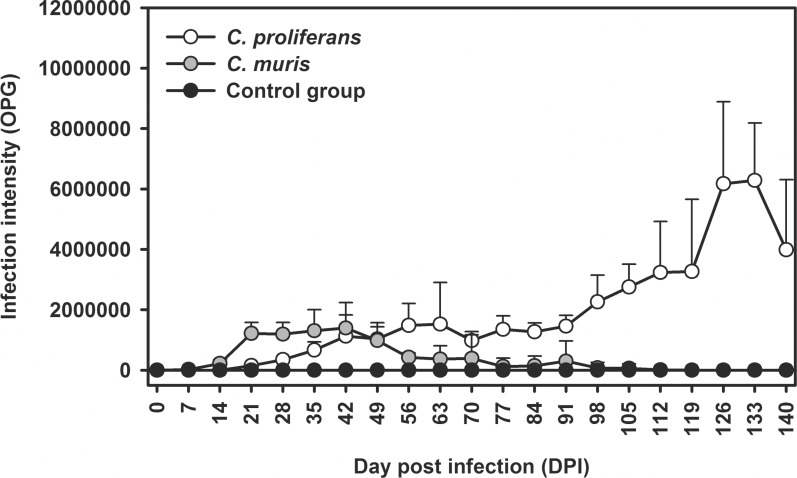
Course of infection of *Cryptosporidium proliferans* and *Cryptosporidium muris* HZ206 in *Mastomys coucha* based on coprological examination of feces.

*Cryptosporidium muris* HZ206 infection intensity ranged from 2.0×10^3^ to 1.4×10^5^ OPG, with maximum shedding at 42 DPI. Oocyst shedding intensity decreased from 42 DPI and microscopic detection was intermittent by 77 DPI, although specific DNA was present in feces throughout the patent period (Fig 7). *Cryptosporidium proliferans* infection intensity ranged from 1.0×10^5^ to 9.5×10^6^ OPG with maximum shedding at 126 DPI. In contrast to *C*. *muris* HZ206, the infection intensity continued to increase throughout the experiment (Fig 7). Southern multimammate mice experimentally infected with *C*. *proliferans* mostly developed lifelong infection (data not shown).

No clinical signs of cryptosporidiosis were detected in southern multimammate mice during the first 84 DPI with *C*. *proliferans*; however, these mice subsequently began to lose weight compared to time-matched mice in the *C*. *muris* HZ206 infection and uninfected control groups (p<0.05).

**Fig 8:**
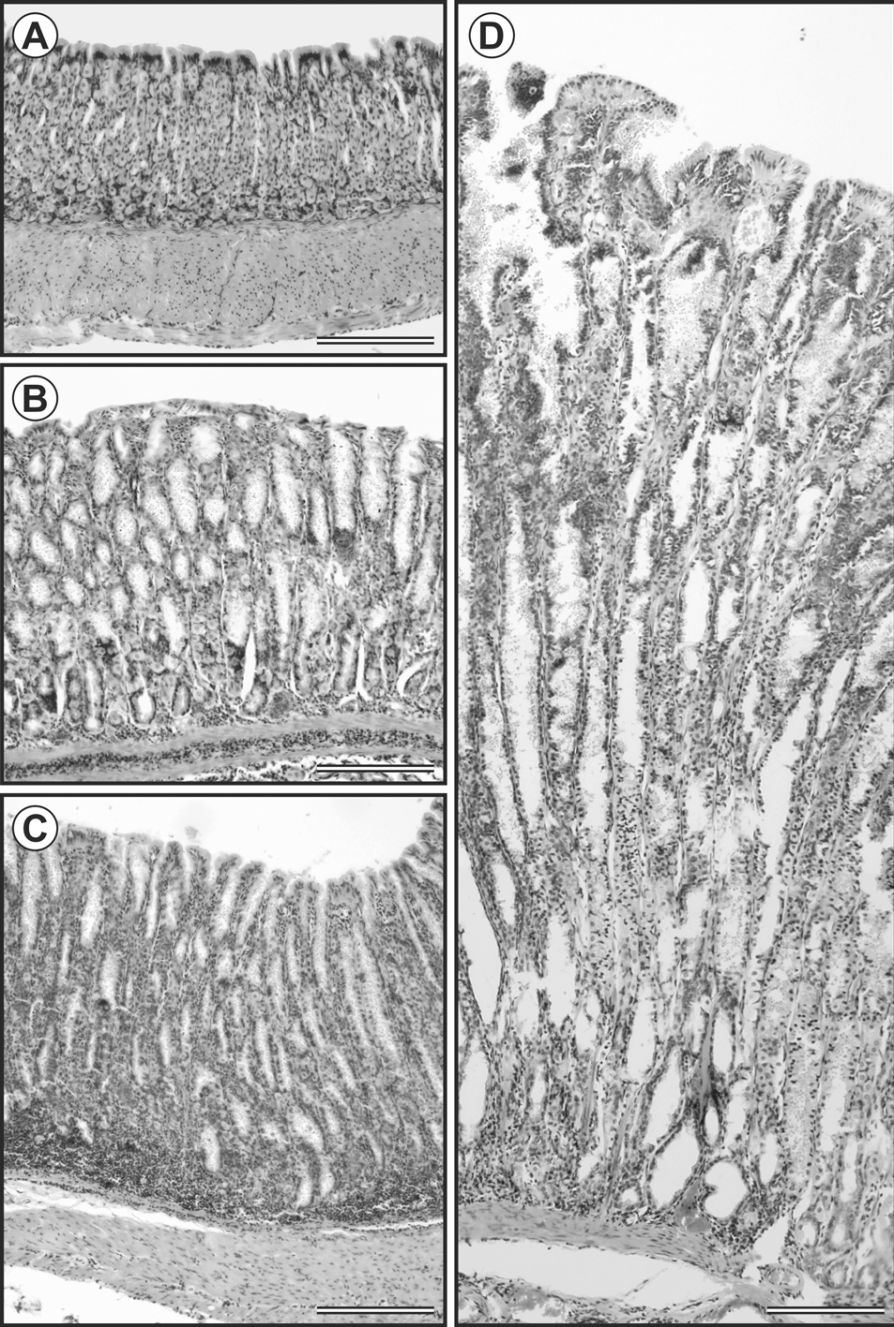
Height of mucosa of *Mastomys coucha* stomach of A) control group, B) *Cryptosporidium muris* HZ206 infection, and C) *Cryptosporidium proliferans* infection at 28 day post infection; D) stomach mucosa proliferation in *Mastomys coucha* with *Cryptosporidium proliferans* at 140 DPI. Haematoxylin and eosin. Bar = 150 μm.

Histological examination of the glandular and non-glandular parts of the stomach in uninfected control mice showed no evidence of *Cryptosporidium* developmental stages, pathological alterations, or activation of glands. Also, mucus production was normal. Developmental stages were found only in the glandular part of the stomach of experimentally infected groups and high numbers were typically associated with high oocyst shedding. In *C*. *proliferans* positive southern multimammate mice, infected glands were markedly dilated, and filled with necrotic material, mucus, and numerous development stages. Epithelial cells of infected glands were atrophic and exhibited cuboidal or squamous metaplasia. The epithelium also was significantly proliferated into the lumen of the stomach and formed papillary structures. Such proliferation was not observed in uninfected control and *C*. *muris* HZ206 infected southern multimammate mice (Fig 8).

**Fig 9:**
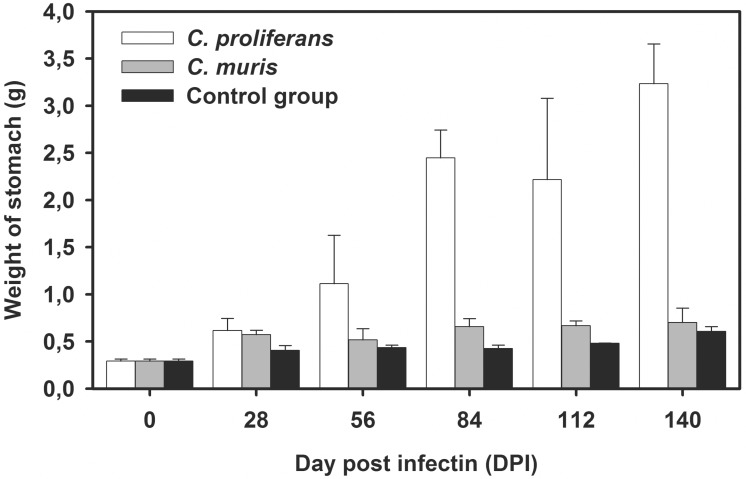
Change of stomach weight of *Mastomys coucha* during experimental infection with *Cryptosporidium proliferans* and *Cryptosporidium muris* HZ206 compared to the control group.

Macroscopically, the gastric mucosa of southern multimammate mice infected with *C*. *proliferans* had confluent cauliflower-shaped lesions (Fig 8D). The *lamina propria* did not contain any inflammatory infiltrates. The gastric mucosa of *C*. *muris* HZ206 infected southern multimammate mice was less hyperplastic without significant gain. Mucus production was similar to that in the uninfected control group and was significantly less than in *C*. *proliferans* infected southern multimammate mice. A gradual retreat of infection from the lower layers of the epithelium was observed in southern multimammate mice infected with *C*. *muris* from 56 DPI, and no developmental stages of *C*. *muris* HZ206 were detected from 112 DPI using histological methods. The total stomach weight in infected southern multimammate mice was increased compared to the negative control group. While the stomach weight of southern multimammate mice infected with *C*. *muris* HZ206 increased up to 1.5 fold, the stomach weight of mice infected with *C*. *proliferans* significantly increased up to 5.7 fold (p<0.001; Fig 9). The proliferating mucosa was the major contributor to the increase in stomach weight, while the submucosa, muscularis, and serosa did not significantly change in any group (p>0.05). The mucosa from *C*. *proliferans* and *C*. *muris* infections increased 5.6 and 1.6 fold, respectively compared to the uninfected control group (p<0.001). In addition, the ratio of glandular to non-glandular surfaces changed from 55:45 to 80:20 in southern multimammate mice infected with *C*. *proliferans*. This change was not observed in uninfected control and *C*. *muris* HZ206 infected southern multimammate mice (data not shown).

### Taxonomic summary of *Cryptosporidium proliferans* n. sp.

#### Diagnosis

Oocysts are shed fully sporulated. Sporulated oocysts 6.8–8.8 (mean = 7.7 μm) × 4.8–6.2 μm (mean = 5.3) with a length to width ratio of 1.48. Four sporozoites and residual body are present in each oocyst. The lifecycle of *C*. *proliferans*, including the description of endogenous stages, was described previously [23].

#### Type host

Tachyoryctes splendens (Rüppell, 1835).

#### Other natural hosts

*Equus africanus* (Fitzinger, 1857), donkey [53]; *Sciurus carolinensis* Gmelin, 1788, eastern gray squirrel [38]; *Syncerus caffer* (Sparrman, 1779), African buffalo [52]; *Equus caballus* Linnaeus, 1758, horse (unpublished, and this study).

#### Experimental host

*Mastomys coucha* (Smith, 1836), southern multimammate mouse; *Mastomys natalensis* (Smith, 1834), natal multimammate mouse; *Meriones unguiculatus* (Milne-Edwards, 1867), Mongolian gerbil; *Lasiopodomys brandtii* (Radde, 1861), Brandt's vole; *Mus musculus* Linnaeus, 1758, house mouse (strain BALB/c and SCID) [15, 22, 24, 25].

Prepatent and patent period of *C*. *proliferans* in model hosts are presented in Table 3.

**Table 3:** Prepatent and patent period of *Cryptosporidium proliferans* in various hosts.

Host	Prepatent period (DPI)	Patent period (DPI)	Reference
**southern multimammate mouse**	15–21	> 140	This study; [24]
**laboratory mouse**	6–8	up to 30	[25]
**gerbils**	18–22	> 90	[21]
**voles**	14	> 40	[24]

#### Type locality

Kakamega, Kenya

#### Site of infection

stomach, specifically the glandular part

#### Material deposited

A phototype, description of oocysts, and DNA are deposited at the Institute of Parasitology, Biology Centre of the Academy of Sciences of the Czech Republic.

#### DNA sequences

Partial sequences of SSU, actin, COWP, HSP70, TRAP-C1, MS1, MS2, MS3, and MS16 genes were submitted to GenBank under the accession numbers KR090615-KR090623 and KT731193-KT731212.

#### Etymology

The species name *proliferans* is derived from the Latin substantive “proliferatio” (meaning a proliferation) according to ICZN Article 11.9.1–3, as it appears to cause a proliferation of mucosa in infected stomach.

## Discussion

The gastric species *C*. *muris* and intestinal species *C*. *parvum* were the first described *Cryptosporidium* species [3, 76]. Until the late 1990s, many intestinal *Cryptosporidium* spp. were regarded as subtypes of *C*. *parvum*; for example, *C*. *hominis* was known as *C*. *parvum* genotype I and *C*. *canis* as *C*. *parvum* canine genotype [77, 78]. Recognition of the subtypes as separate species was made possible by studies showing molecular and biological differences from *C*. *parvum*. In cases where only molecular differences are described, isolates are regarded as distinct genotypes rather than subtypes of *C*. *parvum*; for example, *Cryptosporidium* rat genotype I-IV [79–82]. Also, *C*. *andersoni* (previously known as *C*. *muris*-like) was separated from *C*. *muris senso lato* based on molecular and biological differences [4]. A number of reports over the past 15 years indicate the presence of cryptic species within the mammalian gastric *Cryptosporidium* group, but evidence to support the naming of a new species has thus far been lacking (see Tables 1 and 2 for references).

Although *C*. *muris* and *C*. *andersoni* are primarily rodent- and ruminant specific, respectively, there have been reports of *C*. *muris* in ruminants and *C*. *andersoni* in rodents (Table 1). Similarly, *C*. *proliferans* has been found in hosts belonging to the Rodentia, Arctiodactyla, and Perissodactyla. With the exception of the occurrence of *C*. *proliferans* in squirrels in the US, all the other isolates have been detected in animals in Africa [22, 38, 52] and Europe (this study).

The present study and a previous study [83] have shown that *Cryptosporidium proliferans* oocysts (7.7 × 5.3 μm) are longer and narrower than those of *C*. *muris* HZ206 (7.5 × 5.7 μm) and *C*. *andersoni* (7.6 × 5.5 μm). We have seen no change in the size of the oocysts during more than 10 years of oocyst passage through different hosts (data not shown). However, because the reported size of *C*. *andersoni* and *C*. *muris* oocysts is quite variable [4, 26, 28], oocyst morphology cannot be used to reliably distinguish these species from *C*. *proliferans*. The shape and size of *C*. *proliferans* is also significantly different from oocysts of intestinal species such *C*. *xiaoi* (3.94 × 3.44 μm), *C*. *parvum* (5.3 × 4.7 μm) or *C*. *suis* (6.2 × 5.5 μm) [84, 85].

Phylogenetic analyses based on SSU, COWP, TRAP-C1, HSP70, actin, MS1, MS2, MS3, and MS16 gene sequences have shown that *C*. *proliferans* is genetically distinct from known *Cryptosporidium* species. At the SSU locus, *C*. *proliferans* is 99.4% and 98.3%, similar to *C*. *muris* and *C*. *andersoni*, respectively. This is comparable to the similarities between *C*. *hominis* and *C*. *cuniculus* (98.9%), *C*. *parvum* and *C*. *erinacei* (99.5%), and *C*. *bovis* and *C*. *xiaoi* (99.5%). At the COWP locus, *C*. *proliferans* is 99.7% and 97.8% similar to *C*. *muris* RN66 and *C*. *andersoni*, respectively. Other closely related *Cryptosporidium* species, including *C*. *erinacei* and *C*. *parvum* and *C*. *cuniculus* and *C*. *hominis*, are identical at the COWP locus [86, 87]. At the TRAP-C1 locus, *C*. *proliferans* is 99.0% and 95.6% similar to *C*. *muris* RN66 and *C*. *andersoni*, respectively. In comparison, *C*. *parvum* and *C*. *erinacei* are 99.8% similar at the TRAP-C1 locus [86, 88]. At the HSP70 locus, *C*. *proliferans* is 99.5% and 97.1% similar to *C*. *muris* RN66 and *C*. *andersoni*, respectively. In comparison, *C*. *parvum* and *C*. *erinacei* are 99.0% similar at this locus. *Cryptosporidium proliferans* is identical to the *C*. *muris* Kawatabi isolate (AY643490) at the HSP70 locus [89]. The *C*. *muris* Kawatabi isolate also clusters with *C*. *proliferans* at the SSU locus, suggesting that these could be the same species. Further genetic and biological characterization of the Kawatabi isolate would be required to test this. At the actin locus, *C*. *proliferans* is 99.4% similar to *C*. *muris* RN66. In comparison, *C*. *parvum* and *C*. *erinacei* share 99.5% similarity at this locus. *Cryptosporidium proliferans* also is distinguishable from *C*. *muris* and *C*. *andersoni* at four minisatellite loci examined in this study. It was previously found that *C*. *muris* isolates RN66 and CB03 were identical to *C*. *proliferans* (*C*. *muris* TS03 in their study) at the SSU and minisatellite loci [19]. RN66, a commercially supplied reference strain (Waterborne Inc, LA), has been well characterized and the whole genome sequence is known. Multiple studies have shown that the SSU sequence of this strain differs from that of *C*. *proliferans* (TS03). Similarly, previous studies have shown that CB03 is identical to RN66, and different from TS03, at the SSU locus [22, 25]. Therefore, Feng et al. acknowledged that their finding was likely the result of cross contamination during isolate handling [19].

The prepatent period for *C*. *proliferans* infection in SCID mice, which lack T- and B-cell immunity, ranged from 12–18 DPI with a mean of 14 DPI [20]. This is similar to the prepatent period for *C*. *andersoni* Kawatabi (14 DPI) and longer than the prepatent periods for *C*. *muris* RN66 (6 DPI) and *C*. *muris* CB03 (7 DPI; unpublished) in SCID mice [35]. In a study on *C*. *muris* RN66 infection in nude mice, which specifically lack T-cell immunity [90], a 10 DPI prepatent period was reported following a dose of 1 million oocysts, the dose used in the present study. Longer prepatent periods were observed only with lower doses of 20,000 oocysts (15–18 DPI) and 400 oocysts (16 DPI). Similarly, a prepatent period of 10 DPI was observed in both outbred nude and SCID mice infected with 500,000 oocysts of *C*. *muris* RN66 [91]. Consistent with observations in immunocompromised mice, the prepatent period for *C*. *proliferans* (15–20 DPI) in immunocompetent southern multimammate mice is similar to that for *C*. *andersoni* (20 DPI), and longer than that for *C*. *muris* (6–10 DPI). Collectively, these data support the conclusion that *C*. *proliferans* has a longer prepatent period than *C*. *muris* in mice.

*Cryptosporidium proliferans* develops exclusively in the glandular part of the stomach, similar to *C*. *muris* and *C*. *andersoni* [4, 47, 75, 92], and its lifecycle [23] is similar to that of *C*. *muris* [93].

The clinical course of *C*. *proliferans* infection in immunocompetent southern multimammate mice is considerably different to that of *C*. *muris*. These mice shed oocysts of *C*. *proliferans* for much longer and at a greater intensity than oocysts of *C*. *muris*, and only the *C*. *proliferans* infection causes significant clinical and pathological changes, including weight loss and massive proliferation of the gastric mucosa that is associated with an almost 6-fold increase in stomach weight. Although gastric cryptosporidia rarely induce clinical symptoms in mammals [37, 75, 92, 94], *C*. *andersoni* infection in cattle has caused up to a three-fold increase in abomasum weight, decreased milk production, and loss of body condition [40, 95]. *Cryptosporidium andersoni* also causes a lifelong infection in cattle and mice [4, 22, 96], similar to *C*. *proliferans* in multimammate mice.

Consistent with most previous studies on *C*. *muris* and *C*. *andersoni*, infiltrates in propria of the mucosa are absent in animals infected with *C*. *proliferans* [22, 29, 37, 75, 92–94].

In conclusion, molecular and biological data support the establishment of *C*. *muris* TS03 as a new species and we propose the name *Cryptosporidium proliferans*.
